# PTSD, depression and anxiety among former abductees in Northern Uganda

**DOI:** 10.1186/1752-1505-5-14

**Published:** 2011-08-26

**Authors:** Anett Pfeiffer, Thomas Elbert

**Affiliations:** 1 Department of Psychology, University of Konstanz, Box D23, 78457 Konstanz, Germany

## Abstract

**Background:**

The population in Northern Uganda has been exposed to extreme levels of traumatic stress and thousands abducted forcibly became rebel combatants.

**Methods:**

Using structured interviews, the prevalence and severity of posttraumatic stress disorder (PTSD), depression and anxiety was assessed in 72 former abducted adults, 62 of them being former child soldiers.

**Results:**

As retrospective reports of exposure to traumatic stress increased, anxiety and PTSD occurrence increased (r = .45). 49% of respondents were diagnosed with PTSD, 70% presented with symptoms of depression, and 59% with those of anxiety. In a multiple linear regression analysis four factors could best explain the development of PTSD symptoms: male respondents (sex) living in an IDP-Camp (location) with a kinship murdered in the war (family members killed in the war) and having experienced a high number of traumatic events (number of traumatic events) were more likely to develop symptoms of PTSD than others. In disagreement to a simple dose-response-effect though, we also observed a negative correlation between the time spent with the rebels and the PTSD symptom level.

**Conclusions:**

Former abductees continue to suffer from severe mental ill-health. Adaptation to the living condition of rebels, however, may lower trauma-related mental suffering.

## Background

Humans are developing in a co-constructive way whereby the biological-genetic interface interacts with the cultural setting to form mind and brain and with it the potential for mental malfunctioning. Traumatic stressors evoke an alarm response, i.e., activate stages in a genetically prepared biological defence mechanism that thus appears in any culture. Research into the neurobiological foundations of traumatic experiences [1,2] and data reporting similarity in trauma-related symptom profiles across different cultural settings [3-5] suggest that posttraumatic stress disorder (PTSD) and depression are possible ways of conceptualising mental suffering in response to traumatic stress experiences. Thereby, the cumulative exposure to traumatic experiences, especially when event types vary, seems to have a potentially devastating consequence for mental health, [6,4-13], probably because the exposure to varying types of stressors is particularly powerful to enlarge the fear network [14]. In the age of "new wars" [15], even civilians living in crisis regions are affected by organised violence and human rights violations and often have experienced and witnessed a whole trauma package. Data of Neuner and colleagues [16] for instance, indicate that a two-dozen of traumatic experiences is sufficient to traumatise 100% of any exposed sample.

### War background

In the Northern provinces of Uganda, since 1986, there has been a brutal and unrelenting war, led by a rebel army that named itself the Lord's Resistance Army (LRA). For 17 years members of the LRA have killed or mutilated thousands of innocent civilians and a significant proportion of children have been abducted. According to our own surveys in the camps of internally displaced people (IDP) in Gulu and Kitgum districts, nearly every other boy has been abducted, sometimes only for a few days, to help carry the stolen goods to the bush. Through analysing a database of more than 25.000 children who had been registered in a reception centre after returning from captivity, it can be estimated that 25.000 up to 38.000 children have been abducted between 1986 and 2006 with an average abduction time of 342 days [17]. Many of the young boys, however, have been forced to stay for years, being abused as child combatants while girl child soldiers are regularly abused as sexual slaves [18]. The fear of being terrorised or caught up in the fighting between the LRA and the Ugandan army has caused most of the people to seek refuge in insecure camps with little food and poor sanitation (IDP camps). At the time of the present investigation, about 1,4 million people have been displaced in the affected areas of Northern Uganda. At that time, thousands of people, mainly women and children, marched into the towns and camps seeking shelter, for fear of abduction if they remained in their homes.

The majority of IDPs, currently settled in the country's northern emergency camps, has suffered or witnessed at least one, often several, traumatic experiences. The percentage is especially high within the group of formerly abducted children and young adults. Trauma-related illness compromise vital functioning and thus severely interferes with the ability of refugees and forced migrants to cope with the misery in IDP camps and also limits the capability in rebuilding their homes and lives, regaining ownership and dignity [19].

A cross-sectional epidemiological study done by Roberts [20] among adults living in IDP-camps in Northern Uganda show a high exposure to traumatic war experiences resulting in 54% of PTSD and 67% depression, even with a higher risk among women. Correlating data of Klasen et. al [21] of a strong relationship between traumatic exposure and mental health outcomes could also be found among formerly abducted children. Two other studies both with children still residing in a reception centre showed similar results in trauma exposure and trauma-related responses: up to 10 respectively 11 traumatic war experiences were leading to 35% resp. 38% of moderately to severely traumatic reactions in respect to PTSD [22,23].

Judith L. Herman [24] had defined complex traumatic exposure as being severe in its nature, continuing repeatedly over a long period of time and with an onset during the person's childhood. All of these criteria obviously apply to the experiences of formerly abducted children and young adults. In the present investigation we wanted to further study the severity and frequency of trauma-related mental suffering, particularly of those who have been abducted and specify the relationship between length of abduction as a measure of cumulative trauma exposure and mental health.

## Methods

### Subjects

In May and June 2005, 72 interviews were performed with formerly abducted persons of the Gulu district in Northern Uganda. A minimum of 17 years of age was required for participation. The medium age was 23.7 years. The sample (n = 72) was recruited from two different locations: 42 respondents (20 female) were living at the time of interview in a Reception Centre (RC), having only escaped the rebel movement within the last few weeks. Thirty persons (11 female) living in IDP-camps participated in a follow-up program. The participants were randomly selected from a complete list of persons living in the reception centres at the moment of the study and from a list of people who had already returned home to the IDP-camp after leaving the reception centre (former beneficiaries of the reception centres). Almost all participants belonged to the ethnic group of the Acholi (94%) and all completed the full interview.

Level of education was significantly lower for women than for men, with a third (32%) of the female sample having no education at all. In contrast, nearly two third of the men (61%) had at least a primary education. Men were abducted significantly more often than women, whereas abduction duration was significantly longer than for girls than for the boys (average of 7.7 years vs. 4.9 years). There was no significant difference between the different interview locations (Reception Centre vs. IDP-Camps) for any of the demographic variables.

### Instruments

The questionnaire included a consent form, socio-demographic questions about the person, his/her family, educational level, ethnicity, religion, socioeconomic information, abduction time, trauma experiences, chronic diseases and physical conditions.

Traumatic event types were assessed using a checklist consisting of possible non-war related traumatic event types (forced marriage, witnessing suicide, flood, etc.), war related events (witnessing or experiencing injury by weapon, experiencing an ambush or combat situation, etc.) and LRA-specific traumatic event types (abduction, beatings, torture, forced to beat to death, sexual slavery, forced to maim others, etc.). The checklist was partly taken from the survey "Demography of forced migration" assessed among Sudanese and Ugandan Nationals in the West Nile region [6,16] and compiled by interviewing local victims of the LRA-rebels in order to receive information about their unique atrocities against the civilian population. Events included 19 experienced events, eight witnessed events and three events as forced perpetrator.

PTSD was assessed using an interview Acholi version (Luo language) of the interview form of the Posttraumatic Stress Diagnostic Scale (PDS) [25]. The PDS or its interview form (PSSI) is a widely-used screening instrument for diagnosis and severity of PTSD based on the 17 DSM-IV criteria. Translations in other languages as well as the use in other cultures has been extensive [16,26-28]. The used Acholi version has been also validated for the Luo language within the Acholi culture [29].

For the assessment of depression and anxiety the 25 items from the short version of the "Hopkins Symptom Checklist" (HSCL-25) was used [30]. This screening instrument assesses ten anxiety symptoms and 15 depression symptoms. The scale has been translated in other languages [31,32] and applied in refugee populations [13,33-37]. To identify cases of depression a cut-off score of 1.75 has mostly been used [31,32,20,12]. In a later study, Mollica et al. [38] changed to a scoring algorithm by introducing a DSM-IV based scoring system. Bolton et al. [39] further adapted and refined this algorithm for a study in Rwanda.

The questionnaire for this assessment was translated from the original English version into the local language Luo using the blind back-translation method by Flanagan [40]. The initial translation was accomplished by two trained screeners, thus the translators were not only knowledgeable about local expressions of psychological suffering, but also about clinical diagnostics, procedures and concepts.

Informed consent was obtained using a standardised form explaining the potential risk of participation and explaining that no compensation would be provided. Informed consent forms were signed by the interpreters assuring that s/he has read everything to the respondent and s/he did fully understand their rights. No financial incentives were provided and respondents were informed that no improvements in living conditions were to be expected as a result of participating in the survey. The study was approved by the Konstanz University Ethical Review Board and took place in cooperation with the World Vision Reception Centre for children, for men and child mothers.

## Results

**Figure 1:**
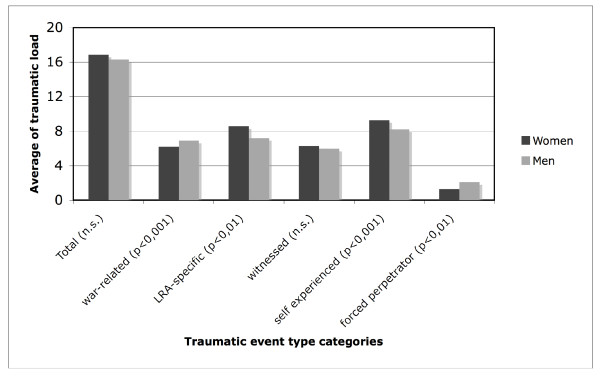
**Average load of traumatic event types by event type category and gender**.

All interviewees had been abducted by LRA rebels and all of them have experienced a series of different traumatic event types, (average 16.5 SD 2.7, from 26 possible event type categories (see Figure 1); table 1 presents an overview of traumatic events that more than 70% of all respondents have experienced or witnessed.

**Table 1:** Traumatic events (experienced or witnessed) by more than 70% of the respondents

	Frequency in % (n = 72)
combat situations (armed attacks, ambushes, fighting, crossfire)	99

being forced to fight (with firearms against UPDF or SPLA)	85

witnessing killing or murder (shot during combat, beaten to death, hacked to death with pangas, axes, sticks or knives)	97

witnessed gunshot wound during combat	99

beatings with sticks, logs or pangas for punishment or initiation rituals	93

witnessed beatings	94

witnessed abduction of a first grade family member**	90

witnessed mutilations	82

being forced to carry heavy loads with threat of death for dropping	94

being threatened to death (e.g. for failed escape attempts)	94

fear of starving or dying of thirst	92

sexual abuse by a stranger (incl. rape, attempted rape, "given as wife" to a LRA rebel commander, being raped by UPDF)	97*

giving birth of a child during abduction (with beatings for screaming and no midwifery assistance)	74*

* only percentage of women (n = 31)

** First grade family members are those who are either directly related by blood in the first degree (parents, children, siblings) or related by marriage (husband, wife). We choose this definition in order to obtain valid data on the status of the immediate affected family members.

Almost half of all respondents (49%) were diagnosed with PTSD meeting the DSM-IV criteria. Data indicate that the prevalence of PTSD is higher in the IDP camps than when still in the reception centre. Such an effect is not observed for the women (see table 2).

**Table 2:** PTSD Diagnosis in percentage (%)

		BY LOCATION		
		RC* (n = 42)	IDP-camp** (n = 30)	**Total** **(n = 72)**

BY GENDER	Men (n = 41)	36	68	51
	
	Women (n = 31)	45	45	45

	Total (n = 72)	40	60	49

* Reception Centre

** Internally displaced persons camp

The depression cut-off score for clinical relevance was reached by 71% of the interviewees and for anxiety by 60%. More than a third of the respondents (36%) fulfil the criteria for all three ascertained mental health disorders simultaneously.

**Figure 2:**
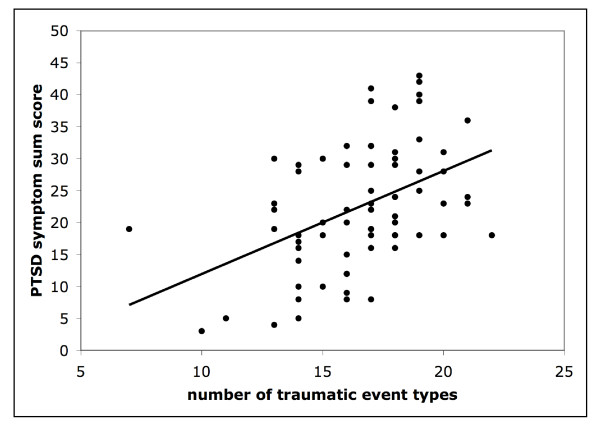
**Scatter plot of the number of traumatic event types and the PTSD symptom sum score with fitted linear regression line**.

The total amount of trauma event types correlates significantly with the PTSD sum score (r = .45, p < 0,001; Figure 2). Looking only at LRA-specific trauma event types, meaning traumatic events that are specifically cruel and unique to the war in Northern Uganda (e.g. witnessed mutilations of lips, ears, noses), and events that describe actions where the person was forced to become a perpetrator and to harm others, also produced significant positive correlations (r = .33, p < .01 and respectively r = .28, p < .05). In addition, the more family members (first grade only) had been killed during the violent clashes the higher the PTSD symptom sum score (r = .24, p < .05). None of the above-mentioned factors, which correlate positively with the posttraumatic symptoms, correlate with the depression symptoms.

**Figure 3:**
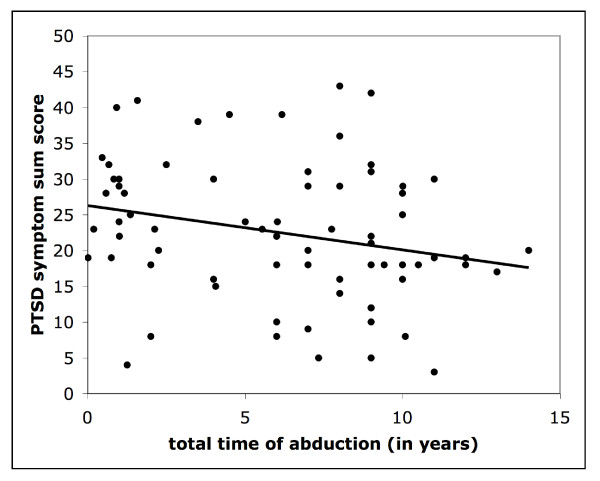
**Scatter plot of the total time of abduction in years and the PTSD symptom sum score with a fitted linear regression line**.

The age at the first time of abduction does not correlate with the posttraumatic symptom score (.056, n.s.). Unexpectedly and in contrast to an expected dose- or "building-block"-effect [16] of exposure to traumatic stress, the duration of the abduction time spent in bush correlates negatively with the PTSD symptom sum score (see Figure 3; r = -.28, p < .05), meaning that the longer a person was abducted, the fewer symptoms were reported.

Using a multiple linear regression, four factors can best explain the PTSD symptom sum score as dependent variable (see table 3): the total number of types of traumatic stressors experienced, the location of living at the time of the interview (Reception Centre vs. IDP-Camp), the amount of killed family members and the sex of the respondent (corrected r-square: .409, F = 13.28, p < .001). In other words: male respondents living in an IDP-Camp with a kinship murdered in the war and having experienced a high number of traumatic events were more likely to develop symptoms of PTSD than others.

**Table 3:** Multiple linear regression with the dependent variable of the PTSD sum score

Model	r	r-square	corrected r-square	Statistic	p
1	.665	.442	.409	F = 13.28	< 0.001

Model 1 - Controlled variables: total amount of all traumatic event types, location, amount of killed family members, sex

## Discussion

The war-affected and formerly abducted young women and men from Northern Uganda who have been interviewed in this study are suffering from severe mental health consequences of the trauma-spectrum disorders (49% diagnosed with PTSD, 71% with symptoms of depression, 60% with those of anxiety) resulting from a high number of experienced traumatic events (in average 16.5 traumatic event types per person). The reported atrocities that more than 70% of the interviewees experienced (see Table 1) took place during the interviewees' forced abduction time with the rebels of the Lord's Resistance Army (LRA) and they quantify descriptions of atrocities documented by human rights reports [41].

The interviewed sample shows to be highly affected by traumatic war experiences and their consequences: almost every other person suffers from PTSD. Similar psychodiagnostic results can also be found in other studies conducted in Northern Uganda [20,21,42] or similarly war torn regions over the world [9,43-45].

PTSD, however, is not the only psychiatric condition that may develop in the aftermath of trauma. On the contrary, comorbidity is the norm rather than the exception. Breslau [46] for example, found that 83% of her PTSD sample met criteria for at least one other psychiatric disorder, compared with 44% of those without PTSD. The National Comorbidity Survey [47] reported that 88% of men and 79% of women with chronic PTSD met criteria for at least one other psychiatric diagnosis. In each of those studies, major depression was found to be one of the most prevalent conditions occurring concurrently with PTSD.

Also the present interviewed sample is not only highly affected by PTSD but shows high symptom scores of depression and anxiety. Evenly high rates of depression were also found in similar war-torn regions, for example among Cambodian refugees with a comparable experience of traumatic events: Carlson & Rosser-Hogan [33] found a rate of 86% PTSD and 80% of clinical depression. This has been equally found for the comorbidity between PTSD and anxiety [13].

There is a significant positive correlation between the amount of experienced traumatic event types and the prevalence of a PTSD. This is coherent with other studies investigating the consequences of organised violence [10-12]. Also the correlation between the exposure to traumatic stressors and symptoms of PTSD is higher than the correlation between traumatic event load and symptoms of depression, since the presence of at least one traumatic event is not only a prerequisite for the diagnosis of PTSD, but also the existence of a dosage relationship has been shown [45,48-50].

Since the amount of traumatic event types is a good predictor for the severity of the symptoms, the specified dose effect can be confirmed. Again, this is consistent with refugee studies from war regions [12,13,45].

If one looks at different levels of traumatisation, the severity of traumatic events can be described using different characteristics such as age of first traumatic event, the intentionality of an event (natural catastrophes vs. human-made deliberate, violent actions), and the duration respectively the re-occurrence of events. As for the traumatic events experienced by the present sample of this study, the classic definition for complex traumatic events postulated by Judith L. Herman [24] seems to well fit their nature as they fulfil all criteria of this definition: The reported traumatic experiences can be rated as highly traumatic due to their violent and cruel nature; they take place over a long period of time (medium duration of abduction: 6 years); they happened repeatedly to the abductees (many of the traumatic events were part of their daily lives); and the interviewees were in average only 14 years old at the time of their first abduction. The experience of traumatic event types, which are infamous for their cruelty and thus severity of traumatic experience (LRA-specific events, events of forced perpetrators and the loss of first grade family members) correlate - in accordance to this definition - positively with the score of posttraumatic symptoms. However none of the other factors - for which it was possible to be ascertained in this study - correlate positively or at all with the posttraumatic stress symptoms. The total amount of experienced traumatic events has not been asked for, as it is difficult or impossible for the interviewees to remember and to count for how often a certain event took place over a period of years. The age at the first time of abduction shows no correlation with the posttraumatic symptoms.

Surprisingly, the duration spent in abduction is correlating negatively with the sum score of the PTSD symptoms. The longer a person is abducted, the lower the sum score of the symptoms gets, but still within a clinical significant range. One possible explanation would be that those who have greater symptoms are more likely to be killed in the bush, or more likely to escape. While we cannot completely rule out this possibility, it would require that fatalities were even higher than the worst estimates. Obviously, survivors who stayed in the bush have adapted to this life with some resilience against PTSD. This adaptation can be argued as a protective and coping mechanism of denial of ongoing horrifying events. Elbert, Weierstall and Schauer [51] though have argued that becoming a perpetrator can be appetitive behaviour disconnecting many of the cues, like for instance "blood" from the neural fear network as they become associated with the fascination for violence and hunting - humans in this case. This pruning of the fear network may result in a decreased vulnerability for PTSD, as has been suggested by recent work [52].

## Conclusions

Even though the sample is limited and not representative for the population of Northern Uganda, it can be shown that children in Northern Uganda - at the time of abduction (mean age of first abduction: 14 years), now youth and young adults (mean age at time of interview: 24 years) - are like other children, youth and adults living in war affected areas are highly affected by the mental health consequences resulting from their violent, cruel and life-threatening traumatic experiences during the war. The psychological suffering of productive symptoms like acted-out flashbacks and active avoidance or "quiet" symptoms like intruding memories or feelings of loneliness thus leads to dysfunctional behaviour in daily routine tasks, social life and work/scholastic life (as dysfunctionality is one criterion to diagnose PTSD). Therefore they are in need of mental health interventions to relieve them from their suffering and make them functional again for their personal daily life tasks as well as in the interest of a society trying to recover from years of insurgencies.

As an interesting tendency that this study's result have shown is the decrease of posttraumatic stress symptoms after a longer time spent in the bush. Although other studies have not found this negative relationship [17] - as this might indicate a survival mechanism, more research is needed in order to find out more about this possibly protective mechanism, whether it can intermediately protect from posttraumatic stress symptoms or may result in other not in this study asked for mental health disorders. Therefore further research is needed on a bigger sample to see if results can be replicated either way. But the focus should not only be on the outcome, but also on the predisposition to this phenomenon.

## Competing interests

The authors declare that they have no competing interests.

## Authors' contributions

AP designed the study, collected the data, performed the statistical analyses and drafted the manuscript. TE supervised the design of the study and the work. All authors participated in revising the manuscript, and have read and approved the final version.
